# An optimization method for hemi-diaphragm measurement of dynamic chest X-ray radiography during respiration based on graphics and diaphragm motion consistency criterion

**DOI:** 10.3389/fphys.2025.1528067

**Published:** 2025-06-26

**Authors:** Yingjian Yang, Jie Zheng, Peng Guo, Tianqi Wu, Qi Gao, Yong Li, Chengcheng Liu, Yang Liu, Yingwei Guo, Huai Chen

**Affiliations:** ^1^ Department of Radiological Research and Development, Shenzhen Lanmage Medical Technology Co., Ltd., Shenzhen, Guangdong, China; ^2^ Department of Medical Image Processing Algorithm, Research and Development Center of Smart Imaging Software, Neusoft Medical System Co., Ltd., Shenyang, Liaoning, China; ^3^ School of Life and Health Management, Shenyang City University, Shenyang, China; ^4^ College of Health Science and Environmental Engineering, Shenzhen Technology University, Shenzhen, China; ^5^ School of Electrical and Information Engineering, Northeast Petroleum University, Daqing, China; ^6^ Department of Radiology, The Second Affiliated Hospital of Guangzhou Medical University, Guangzhou, China

**Keywords:** dynamic chest radiography, hemi-diaphragm measurement, convolutional neural network, graphics, diaphragm motion consistency criterion, respiration

## Abstract

**Introduction:**

Existing technologies are at risk of abnormal hemi-diaphragm measurement due to their abnormal morphology caused by lung field deformation during quiet breathing (free respiration or respiratory) interventions in dynamic chest radiography (DCR). To address this issue, an optimization method for hemi-diaphragm measurement is proposed, utilizing graphics and the consistency criterion for diaphragm motion.

**Methods:**

First, Initial hemi-diaphragms are detected based on lung field mask edges of dynamic chest X-ray images abstracted from the DCR at respiratory interventions controlled by the radiologist’s instructions. Second, abnormal hemi-diaphragms are identified, resulting from morphological deformation of the lung field during respiration. Lastly, these abnormal hemi-diaphragms are optimized based on the consistency criterion of diaphragm motion.

**Results:**

Results show that the proposed optimization method can effectively measure the hemi-diaphragm, even in the presence of the inapparent cardiophrenic angle caused by abnormal deformations of the lung field morphology during respiration, reducing the mean error by 49.050 pixels (49.050 × 417 μm = 20,453.85 μm).

**Discussion:**

Therefore, the proposed optimization method may become an effective tool for precision healthcare to find the pattern of diaphragm movement during respiratory interventions.

## 1 Introduction

X-ray is the most widely used primary imaging modality for routine chest and bone radiography as it is widely available, low-cost, has a fast imaging speed, and is easy to acquire (Liu et al., 2022; Yang et al., 2024a). Specifically, a digital X-ray image can be obtained within seconds after exposure by directly projecting the captured image of the human body onto a two-dimensional plane (Yang et al., 2024a; Yang et al., 2024b). Therefore, it has become the preferred imaging device to improve work efficiency and facilitate the initial chest diagnosis of critically ill and/or emergency patients in clinical practice (Yang et al., 2024a; Yang et al., 2024b; Howell, 2016; Irmici et al., 2023).

As the most widely used static chest imaging modality, the static chest X-ray technique captures the thorax and surrounding structures at a specific moment, resulting in a chest X-ray plain film. The chest X-ray plain film can display the inside and outside structures of the chest cavity, which is most helpful in identifying abnormalities in the heart, lung parenchyma, pleura, chest wall, diaphragm, mediastinum, and hilum (Reed, 2011). Therefore, it is typically used as a preliminary examination to evaluate diseases such as thoracic diseases or damage (such as rib fractures), lung diseases (such as pneumonia and COVID-19), and cardiovascular diseases (such as an abnormal cardiothoracic ratio) (Yang et al., 2024a; Sun et al., 2023; Amin et al., 2024). It effectively alleviates the slow imaging speed of chest computed tomography (CT), magnetic resonance imaging (MRI), and positron emission tomography (PET), especially in clinical practice for critically ill and/or emergency patients (Yang et al., 2024a; Yang et al., 2024b; Howell, 2016; Irmici et al., 2023). However, the static chest imaging modality limits the dynamic analysis of lung physiological activity, such as ventilation (Yang et al., 2025), changes in the cardiothoracic ratio (Yang et al., 2024a), alterations in lung field area, and diaphragm movement during lung respiration (Yang et al., 2024b).

Compared to static chest imaging modalities, dynamic chest functional imaging modalities facilitate quantitative analysis of lung physiological activity from an anatomical perspective. These modalities include chest CT, MRI, X-ray, scintigraphy, PET, ultrasound, and electrical impedance tomography (EIT) for quantitative analysis of lung physiological activity, such as evaluating ventilation and perfusion in specific regions (Nakamura et al., 2024). Additionally, the dynamic chest MRI is particularly valuable for quantifying the severity of chest wall deformation in children with spinal deformities, which is crucial for understanding its impact on trunk appearance and cardiopulmonary function (Arias-Martínez et al., 2025). However, compared to other dynamic chest functional imaging modalities, dynamic X-ray and CT imaging modalities are the preferred choices for diagnosing chest diseases in clinical practice (Al-qaness et al., 2024). Dynamic chest X-ray and CT imaging modalities are not conflicting but complementary, which are crucial in chest medical imaging. Specifically, chest X-ray fluoroscopy, a type of dynamic chest radiography (DCR), is a real-time, sequential, high-resolution digital X-ray imaging system of the thorax in motion over the respiratory cycle, utilizing pulsed image exposure. Post-acquisition image processing by a computer algorithm automatically characterizes the motion of thoracic structures (Fyles et al., 2023). Compared with chest X-ray fluoroscopy, the chest CT sacrifices temporal resolution to obtain higher three-dimensional spatial resolution. Specifically, although the chest CT provides a more precise definition of the structure and abnormalities within the thorax than the chest X-ray fluoroscopy, due to its limitations on radiation dose, chest CT images are currently only acquired at deep exhalation and/or deep inhalation, obtaining inspiratory or/and expiratory chest CT images (Yang et al., 2022a; Deng et al., 2024; Wang et al., 2024). However, the lungs undergo irregular deformation during the respiratory process (Gong et al., 2024). Compared to the inspiratory and expiratory chest CT images (at the two time points), chest fluoroscopy encompasses more time points during quiet breathing (free respiration or respiratory) interventions. Therefore, this significantly contributes to the dynamic quantitative analysis of lung movement function, such as hemi-diaphragm motion.

Specifically, Tanaka et al. assessed the correlation between diaphragm motion parameters and lung vital capacity (Tanaka et al., 2006). Meanwhile, Yamada et al. evaluated the average diaphragmatic excursions in healthy volunteers and the difference in tidal breathing diaphragm motion between COPD and healthy controls using DCR (Yamada et al., 2017b; Yamada et al., 2017a). Subsequently, Yamada et al. further assessed the correlation between diaphragm motion and anthropometrics (Yamamoto et al., 2020). In addition, Hida et al. assessed diaphragm motion in standing positions during forced breathing and evaluated its associations with demographics and pulmonary function tests. Subsequently, Hida et al. further assessed the differences in speed and excursion of diaphragmatic motion between patients with COPD and controls, as well as the correlation between pulmonary function tests and diaphragmatic motion (Hida et al., 2019b; Hida et al., 2019a). Besides, FitzMaurice et al. described the changes in diaphragm motion and lung areas before and after modulator therapy in adults with cystic fibrosis bronchiectasis using DCR (FitzMaurice et al., 2022c). Subsequently, FitzMaurice et al. further described diaphragm motion in individuals with a paralyzed hemi-diaphragm using DCR, as well as diaphragm motion in individuals undergoing treatment for a pulmonary exacerbation of cystic fibrosis bronchiectasis (FitzMaurice et al., 2022b; FitzMaurice et al., 2022a). Additionally, Chen et al. quantitatively evaluated diaphragmatic motion during forced breathing in patients with chronic obstructive pulmonary disease using DCR (Chen et al., 2022). Therefore, precision hemi-diaphragm detection in DCR images is crucial for accurately assessing diaphragm movement function (Yang et al., 2024b).

Based on the above, Yang et al. proposed an effective hemi-diaphragm detection method using a convolutional neural network (CNN) and Graphics for its accurate evaluation (Yang et al., 2024b). This hemi-diaphragm detection method can potentially localize the cardiophrenic angle based on the morphology of the left and right lung field mask edge images, utilizing graphics to assist with hemi-diaphragm measurement. However, the measurement method of the hemi-diaphragm mentioned above often yields an abnormal measurement of the hemi-diaphragm in the left lung field due to its abnormal morphology resulting from lung field deformation in DCR. Therefore, it is necessary to propose an optimization method to ensure the accuracy of hemi-diaphragm measurement on the dynamic chest X-ray (CXR) images for subsequent quantitative analysis. Our contributions in this paper are briefly described as follows:

(1) We propose an abnormal hemi-diaphragm identification method caused by morphological deformation of the lung field motion during respiration, which is crucial for subsequent hemi-diaphragm optimization.
(2) We propose a hemi-diaphragm optimization method based on the diaphragm motion consistency criterion to optimize these abnormal hemi-diaphragms, even if there is an inapparent cardiophrenic angle caused by abnormal deformations of the lung field morphology during respiration.
(3) The proposed optimization method may become an effective tool for identifying the pattern of diaphragm movement during respiratory interventions for precision healthcare.

## 2 Materials and methods

The proposed hemi-diaphragm optimization method involves the initial measurement of the hemi-diaphragm from dynamic CXR images extracted from the DCR, followed by the optimization of the abnormal hemi-diaphragm. Based on the above, materials and methods are described in Sections 2.1 and 2.2, respectively.

### 2.1 Materials

Seven hundred seventy-six static CXR images (512 × 512) predefined by pneumonia, tuberculosis, unclear disease, and health (the normal case in the data description) were collected from public CXR datasets and the Radiopaedia website accessed by Google browser (https://radiopaedia.org/). They were used to train and test the standard lung field segmentation model based on a CNN architecture. The fifteen static Internet CXR images were collected from the Radiopaedia website (https://radiopaedia.org/articles/chest-pa-view-1, https://radiopaedia.org/articles/chest-radiograph?lang=us, and https://radiopaedia.org/articles/chest-expiratory-view-2?lang=us), an open-source, expert-reviewed, and extensive radiology encyclopedia. Detailed information on these 776 static CXR images can be found in our previous research (Yang et al., 2024a; Yang et al., 2024b). In addition, five sets of DCR (the CXR video) at respiratory interventions controlled by the radiologist’s instructions are collected by a digital X-ray imaging system (manufacturer: Lanmage, collection mode: chest fluoroscopy, and flat panel detector: IRAY). Specifically, 30 dynamic CXR images are abstracted from each DCR. Table 1 summarizes the characteristics of these 150 (30 × 5) dynamic CXR images. Specifically, these participants received prior guidance from radiologists on respiratory intervention control, which trained them to breathe quietly in the standing position. Then, they underwent postero-anterior digital X-ray imaging while breathing quietly in the standing position.

**TABLE 1:** Characteristics of these five sets of DCR (150 dynamic CXR images).

Characteristics	Value/Mean ± SD^a^
Gender (male/female)	3/2
Age (year)	41.2 ± 24.964 (Range: 21–69)
kVp	77.0 ± 2.739 (Range: 75–80)
Distance source to the detector (cm)	180
Exposure time (ms)	125
X-ray tube current (mA)	110.0 ± 13.693 (Range: 100–125)
Entrance dose in mGy	0.282 ± 0.208 (Range: 0.130–0.510)
Frames/s	15

^a^ The SD denotes the standard deviation.

Written informed consents were obtained from these participants, and the study was approved by the Guangzhou Medical University Ethics Committee in China (Grant number: 2023-hg-ks-24, Approval Date: 28 August 2023, Tel: +86-20-34153599, Fax: +86-20-34153066).

### 2.2 Methods

**FIGURE 1:**
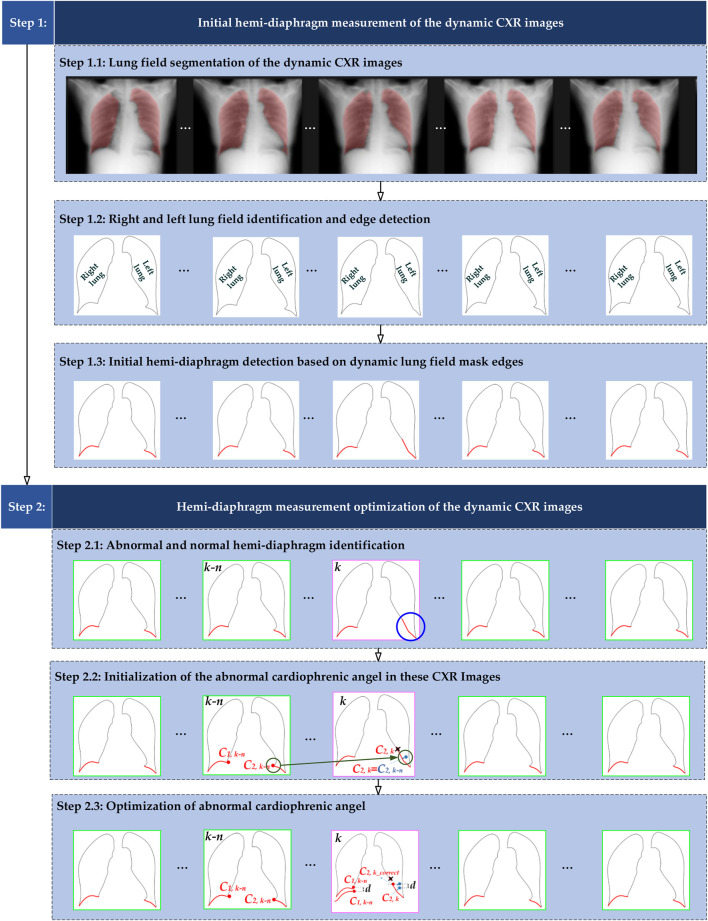
Overall flowchart of the proposed method for optimizing hemi-diaphragm measurement in dynamic CXR images. The pink box: CXR images with an abnormal hemi-diaphragm that requires correction. The green box: CXR images with the normal hemi-diaphragm.

Figure 1 illustrates the overall flowchart of the proposed method for optimizing hemi-diaphragm measurement in dynamic CXR images. Specifically, the proposed hemi-diaphragm optimization method includes two main steps. Step 1 completes the initial hemi-diaphragm measurement of the dynamic CXR images. Subsequently, step 2 completes the optimization of the abnormal hemi-diaphragm based on the measurement obtained in step 1.

#### 2.2.1 Initial hemi-diaphragm measurement

**FIGURE 2:**
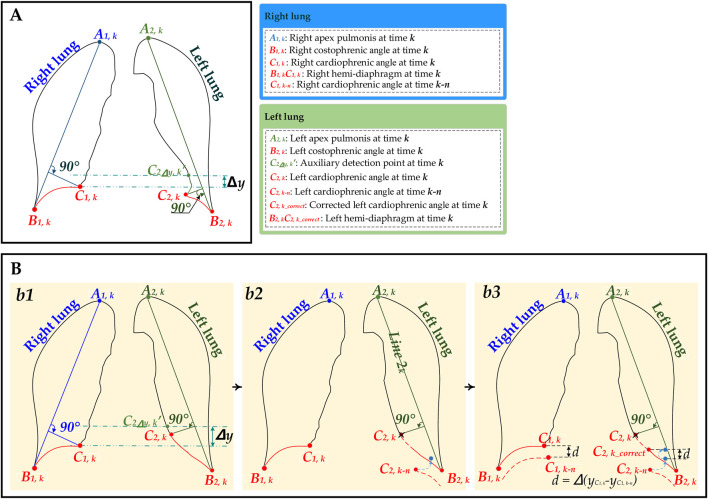
The schematic diagram for the hemi-diaphragm measurement based on left and right lung field mask edge images. **(A)** The initial hemi-diaphragm measurement (normal). **(B)** Optimized process of the abnormal hemi-diaphragm measurement. (b1) The initial hemi-diaphragm measurement (abnormal). (b2) Initialization of the abnormal left cardiophrenic angle. (b3) Optimization of abnormal left cardiophrenic angle.

Figures 1, 2A show that the initial hemi-diaphragm measurement is based on our previous method (Yang et al., 2024b). Specifically, this method localizes the right cardiophrenic angle based on the edge of its lung field mask. Then, the left cardiophrenic angle is localized based on the right cardiophrenic angle and the edge of the left lung field mask. Lastly, the initial right hemi-diaphragm is determined by a line segment extending from the right cardiophrenic angle to the right costophrenic angle along the edge of the right lung field mask. Similarly, the initial left hemi-diaphragm is determined by a line segment from the left cardiophrenic angle to the left costophrenic angle along the left lung field mask edge.

##### 2.2.1.1 Lung field segmentation

A pre-trained, robust, and standard lung field segmentation model is used to abstract the lung field from these 150 dynamic CXR images, generating 30 lung field mask images of each case. Specifically, the organ and lesion segmentation model for medical images based on CNNs has become an indispensable technology for quantitative analysis (Yang et al., 2024a; Zaman et al., 2024; Zeng et al., 2023; Duan et al., 2023; Yang et al., 2021; Junia and K, 2024; Iqbal et al., 2021; Iqbal et al., 2020; Mochurad, 2025). However, a robust and standardized lung field segmentation model for cross-center and pathological CXR images remains to be developed for quantitative analysis based on the lung field.

Based on the above, the metrics of five standard lung field segmentation models based on the fully convolutional networks (FCN) (Long et al., 2015), SegNet (Badrinarayanan et al., 2017), U-Net (Ronneberger et al., 2015), and its two improved networks (ResU-Net++ (Jha et al., 2019) and AttU-Net (Wang et al., 2022)) with these 776 static CXR images and data augmentation technique, were evaluated to validate that automatic lung field segmentation in routine CXR imaging is a data diversity problem, not a methodology problem (Yang et al., 2024b). These networks, including FCN, SegNet, U-Net, ResU-Net++, and AttU-Net, are trained sequentially using the same training set, generating five lung field segmentation models. Then, five standard evaluation metrics, including accuracy, precision, recall, Dice, and Intersection over Union (IoU), as well as the 95th percentile Hausdorff distance (HD), are calculated for these lung field segmentation models using the same test set, respectively (Yang et al., 2024a; Yang et al., 2024b; Yang et al., 2025). Specifically, the mean accuracies (%) of these lung field segmentation models are 98.75 ± 0.49, 98.93 ± 0.63, 98.93 ± 0.85, 99.02 ± 0.60, and 99.05 ± 0.69, respectively. In addition, the mean precision (%) of these lung field segmentation models is 97.56 ± 1.15, 97.89 ± 1.49, 93.30 ± 1.40, 97.80 ± 1.96, and 98.36 ± 1.44, respectively. The mean recall (%) of these lung field segmentation models is 97.14 ± 1.73, 97.55 ± 1.93, 97.31 ± 2.70, 98.05 ± 1.69, and 97.67 ± 2.14, respectively. The mean Dice (%) of these lung field segmentation models is 97.35 ± 1.19, 97.71 ± 1.56, 97.78 ± 1.63, 97.91 ± 1.46, and 97.99 ± 1.43, respectively. The mean IoU (%) of these lung field segmentation models is 94.85 ± 2.22, 95.57 ± 2.91, 95.71 ± 3.05, 95.95 ± 2.74, and 96.11 ± 2.69, respectively. Finally, the mean 95th percentile HD of these lung field segmentation models is 5.61 ± 3.26, 5.41 ± 3.81, 5.72 ± 5.02, 5.46 ± 4.51, and 5.02 ± 4.15, respectively.

Although these evaluation metrics indicate no significant difference between these lung field segmentation models, this lung field segmentation based on FCN was excluded due to the noticeable jagged edges in the lung field masks (Yang et al., 2024a; Yang et al., 2024b; Yang et al., 2025). Meanwhile, due to the simple network structure and limited computing resources of U-Net, the lung field segmentation model based on U-Net is ultimately adopted in this study.

##### 2.2.1.2 Lung field identification and edge detection

The right and left lung fields are identified based on their area in each lung field mask image (Yang et al., 2024b). Additionally, an edge detection algorithm is applied to the lung field mask images, resulting in 30 edge images for each case (Yang et al., 2024b). Specifically, this edge detection algorithm uses a 3 × 3 pixel correction template to traverse each lung field mask image in rows/columns with a step size of 1 pixel to generate the corroded lung field mask image. Then, the lung field mask edge images are obtained by subtracting the corroded lung field mask image from its corresponding uncorroded lung field mask image.

##### 2.2.1.3 Initial hemi-diaphragm detection

The initial left and right hemi-diaphragm are separately measured based on the right and left lung field mask edge images.

Specifically, the right cardiophrenic angles at time *k C*_*1,k*_ of each case are cleverly localized by the maximum Euclidean distance from the straight line at time *k A*_*1,k*_ *B*_*1,k*_ (*k* = 1,2,3, … ,30) shown in the mathematical expressions Equations 1, 2:

$$A_{1,k}B_{1,k}:a_{1,k}x+b_{1,k}y+c_{1,k}=0,$$ (1)

$$\begin{array}{l} {C_{1}}_{,k}\left(x,y\right)\leftarrow \max\vec{D_{C1,k}}\\ =\max\left(d_{r1,k}\left(p_{r1,k}\right),d_{r2,k}\left(p_{r2,k}\right)...,d_{rn,k}\left(p_{rn,k}\right)\right)\\ =\max\left(d_{r1,k}\left(x_{r1,k},y_{r1,k}\right),d_{r2,k}\left(x_{r2,k},y_{r2,k}\right),...,d_{rn,k}\left(x_{rn,k},y_{rn,k}\right)\right)\\ =\max\left(\frac{\left|a_{1,k}x_{r1,k}+b_{1,k}y_{r1,k}+c_{1,k}\right|}{\sqrt{a_{1,k}^{2}+b_{1,k}^{2}}},...,\frac{\left|a_{1,k}x_{rn,k}+b_{1,k}y_{rn,k}+c_{1,k}\right|}{\sqrt{a_{1,k}^{2}+b_{1,k}^{2}}}\right) \end{array},$$ (2)

Where $\vec{D_{C1,k}}=\left(d_{r1,k}\left(p_{r1,k}\right),d_{r2,k}\left(p_{r2,k}\right)...,d_{rn,k}\left(p_{rn,k}\right)\right)$ denotes the *i*th Euclidean distances at time *k* $d_{ri,k}$ of these coordinates $\left(p_{r1,k},p_{r2,k},...,p_{rn,k}\right)=\left(\left(x_{r1,k},y_{r1,k}\right),\left(x_{r2,k},y_{r2,k}\right),...,\right.$ $\left.\left(x_{rn,k},y_{rn,k}\right)\right)$ extracted from all pixels (*A*_*1,k*_ to *B*_*1,k*_) in the right lung edge at time *k* ${Ι}_{mask\_right\_edge,k}$ on the right side of the *A*_*1,k*_ *B*_*1,k*_ to the straight line *A*_*1,k*_ *B*_*1,k*_, and *i =* 1, 2, … , *n*. These parameters $a_{1,k},b_{1,k},c_{1,k}$ denote the coefficients of the straight line *A*_*1,k*_ *B*_*1,k*_, and *r* denotes the right lung.

Subsequently, the left cardiophrenic angle at time *k, C*_*2,k*_ *, is localized based on the right cardiophrenic angle and the* left lung field mask edge.

Specifically, since this left cardiophrenic angle at time *k C*_*2,k*_ is not the farthest point from the straight line *A*_*2,k*_ *B*_*2,k*_ *(k = 1,2,3, … ,30)*, it is necessary to restrict the coordinate points extracted from all pixels in the left lung edge at time *k* ${Ι}_{mask\_left\_edge,k}$ on the left side of the *A*_*2,k*_ *B*_*2,k*_. Therefore, the empirical preset constant parameter Δy (20 pixels) is introduced to restrict the coordinate points far from the left cardiophrenic angle *C*_*2,k*_. The left intersection of the horizontal line $y=y_{C_{1,k}\left(x,y-\Delta y\right)}$ and the left lung edge at time *k* ${Ι}_{mask\_left\_edge,k}$ is configured as an auxiliary measurement point ${C_{2\Delta y,k}}^{\prime }\left(x,y\right)$ . Thus, this left cardiophrenic angle at time *k C*_*2,k*_ is constrained from the edge segment *A*_*2,k*_ *B*_*2,k*_ to the edge segment ${C_{2\Delta y,k}}^{\prime }\left(x,y\right)B_{2,k}$ on the left lung edge ${Ι}_{mask\_left\_edge,k}$ .

The above specific implementation details are represented by mathematical expressions Equation 3–5:

$$A_{2,k}B_{2,k}:a_{2,k}x+b_{2,k}y+c_{2,k}=0,$$ (3)

$$\left\{\begin{array}{l} y=y_{C_{1,k}\left(x,y-\Delta y\right)}\\ {Ι}_{mask\_left\_edge,k}=0 \end{array}\right.\to {C_{2\Delta y,k}}^{\prime }\left(x,y\right),$$ (4)

$$\begin{array}{l} {C_{2}}_{,k}\left(x,y\right)\leftarrow \max\vec{D_{C2,k}}\\ =\max\left(d_{l1,k}\left(p_{r1,k}\right),d_{l2,k}\left(p_{l2,k}\right)...,d_{lm,k}\left(p_{lm,k}\right)\right)\\ =\max\left(d_{l1,k}\left(x_{l1,k},y_{l,k}\right),d_{l2,k}\left(x_{l2,k},y_{l2,k}\right),...,d_{\ln,k}\left(x_{lm,k},y_{lm,k}\right)\right)\\ =\max\left(\frac{\left|a_{2,k}x_{l1,k}+b_{2,k}y_{l1,k}+c_{2,k}\right|}{\sqrt{a_{2,k}^{2}+b_{2,k}^{2}}},...,\frac{\left|a_{2,k}x_{\ln,k}+b_{2,k}y_{\ln,k}+c_{2,k}\right|}{\sqrt{a_{2,k}^{2}+b_{2,k}^{2}}}\right) \end{array},$$ (5)

Where $\vec{D_{C2,k}}=\left(d_{l1,k}\left(p_{l1,k}\right),d_{l2,k}\left(p_{l2,k}\right)...,d_{\ln,k}\left(p_{\ln,k}\right)\right)$ denotes the *i*th Euclidean distances at time *k* $d_{li,k}$ of these coordinates $\left(p_{l1,k},p_{l2,k},...,p_{\ln,k}\right)=\left(\left(x_{l1,k},y_{l1,k}\right),\left(x_{l2,k},y_{l2,k}\right),...,\right)$ $\left.\left(x_{\ln,k},y_{\ln,k}\right)\right)$ extracted from all pixels ( ${C_{2\Delta y,k}}^{\prime }\left(x,y\right.$ to *B*_*1,k*_) in the left lung edge ${Ι}_{mask\_left\_edge,k}$ on the left side of the *A*_*2,k*_ *B*_*2,k*_ to the straight line *A*_*2,k*_ *B*_*2,k*_, and *i* = 1, 2, … , *m*. These parameters $a_{2,k},b_{2,k},c_{2,k}$ separately denote the coefficients of the straight line *A*_*2,k*_ *B*_*2,k*_, and *l* denotes the left lung.

Last, the initial right hemi-diaphragm *A*_*1,k*_ *C*_*1,k*_ is determined by a line segment from the right cardiophrenic angle at time *k C*_*1,k*_ to the right costophrenic angle *A*_*1,k*_ along the right lung field mask edge ${Ι}_{mask\_right\_edge,k}$ . Similarly, the initial left hemi-diaphragm *A*_*2,k*_ *C*_*2,k*_ is determined by a line segment from the left cardiophrenic angle at time *k C*_*2,k*_ to the left costophrenic angle *A*_*2,k*_ along the left lung field mask edge ${Ι}_{mask\_left\_edge,k}$ .

#### 2.2.2 Hemi-diaphragm measurement optimization

Figures 1, 2B show the optimized process of the abnormal hemi-diaphragm measurement based on left and right lung field mask edge images.

##### 2.2.2.1 Abnormal and normal hemi-diaphragm identification

These abnormal and normal hemi-diaphragm images should be identified in the dynamic CXR images abstracted from the same DCR.

The abnormal hemi-diaphragm is often accompanied by morphological deformation of dynamic lung field motion during respiration. This morphological deformation frequently occurs in the left lung field and can result in the inconspicuousness of the left cardiophrenic angle at time *k C*_*2,k*_. Therefore, an abnormal hemi-diaphragm often appears in the lung field, and due to the inconspicuousness of the left cardiophrenic angle at time *k C*_*2,k*_, the length of the abnormal left hemi-diaphragm is longer than that of the normal left hemi-diaphragm. Based on the above, an innovative method for detecting abnormal hemi-diaphragm is proposed.

Specifically, the shortest left hemi-diaphragm of these dynamic CXR images can be determined using this abnormal hemi-diaphragm detection method. Then, the shortest left hemi-diaphragm length is configured as the baseline length $l_{base}$ . Subsequently, the difference between the length of each left hemi-diaphragm and this baseline length is calculated. If this difference exceeds the empirical preset difference length Δl (20 pixels), the left hemi-diaphragm is determined as an abnormal left hemi-diaphragm $hemi_{abnormal}$ . Otherwise, this left hemi-diaphragm is determined to be a normal left hemi-diaphragm $hemi_{normal}$ .

The above specific implementation details are represented by mathematical expressions Equations 6, 7:

$$l_{base}=\min\left(l_{1},l_{2},...,l_{i},...,l_{k}\right),i=1,2,3,...,k,$$ (6)

$$\left\{\begin{array}{l} if\left|l_{i}-l_{base}\right|\geq \Delta l;\to l_{i}\in hemi_{abnormal}\\ else\to l_{i}\in hemi_{normal} \end{array}\right.,$$ (7)

where $l_{i}$ denotes the length of the left hemi-diaphragm at time *i*, and $l_{base}$ denotes the shortest initial left hemi-diaphragm of all initial left hemi-diaphragms (the baseline length). Besides, $hemi_{abnormal}$ and $hemi_{normal}$ separately denote the abnormal and normal left hemi-diaphragm.

##### 2.2.2.2 Diaphragm motion consistency criterion

Several studies have determined reference values for diaphragmatic motion and sought to establish a correlation between diaphragm movement displacement and lung diseases, using dynamic chest images obtained from various imaging modalities, including X-ray (Hida et al., 2019b; Hida et al., 2019a; FitzMaurice et al., 2022c; FitzMaurice et al., 2022b; FitzMaurice et al., 2022a; Chen et al., 2022), ultrasound (Boussuges et al., 2009), and MRI (Hao et al., 2025). Specifically, the displacement values of the right and left diaphragmatic excursions were measured using M-mode ultrasound in 210 healthy adult subjects (150 men and 60 women) at the standing position, providing consistency in the displacement of the right and left diaphragmatic excursions (Boussuges et al., 2009). In addition, the caudocranial displacements of the 25 points from end-expiration (EE) to end-inspiration (EI) were quantified, and the velocity of the surfaces of right and left hemi-diaphragms at each point was separately derived by dividing the displacement from EE to EI by the time interval from EE to EI by dynamic MRI, proving strong correlations in velocity between homologous regions of right and left hemi-diaphragms (Hao et al., 2025). Most notably, the range of hemi-diaphragm excursion observed using DCR is proven to be similar to that observed using M-mode ultrasound (Al-qaness et al., 2024). This illustrates the scientific and rational nature of the diaphragm motion consistency.

Additionally, the underlying cause of abnormal diaphragm detection in the existing technologies is the inapparent cardiophrenic angle on DCR resulting from lung field deformation during respiration. Therefore, it is necessary to consider modifying this abnormal diaphragm based on the timing characteristics of DCR, such as consistency in diaphragm motion at the same time.

Based on the above analysis, the diaphragm motion consistency criterion is proposed to assist in optimizing the abnormal left hemi-diaphragm at time *k C*_*2,k*_. Anatomically, the left and right hemi-diaphragm are at different locations of the same diaphragm. Therefore, the subsequent study assumes that the relative vertical motion displacement *d*_*k*_ of the cardiophrenic angles at time *k C*_*1,k*_, *C*_*2,k*_ and the cardiophrenic angles at time *k ± n C*_*1,k*±*n*_, *C*_*2,k*±*n*_ are consistent. This proposed diaphragm motion consistency criterion is represented by mathematical expressions Equation 8:

$$d_{k}\approx y_{C_{1,k}}-y_{C_{1,k\pm n}}\approx y_{C_{2,k}}-y_{C_{2,k\pm n}},$$ (8)

Where $y_{C_{1,k}}$ and $y_{C_{1,k\pm n}}$ separately denote the right cardiophrenic angles at time *k* and *k ± n* in the *y* direction. Besides, $y_{C_{2,k}}$ and $y_{C_{2,k\pm n}}$ separately denote the left cardiophrenic angles at time *k* and *k ± n* in the *y* direction.

##### 2.2.2.3 Optimization of abnormal cardiophrenic angle

Because the position detection mistakes of the left and right cardiophrenic angles will cause abnormal hemi-diaphragm, the optimization task for hemi-diaphragm measurement is to correct the positions of these left and right cardiophrenic angles. Meanwhile, based on engineering experience and the morphological characteristics of the lung, this situation often occurs at the left cardiophrenic angle.

First, each original left cardiophrenic angle *C*_*2,k*_ of the abnormal diaphragm $hemi_{abnormal}$ to be corrected is initialized separately by the most adjacent left cardiophrenic angle *C*_*2,k*±*n*_ of the normal diaphragm $hemi_{normal}$ . Second, the relative vertical motion displacement *d*_*k*_ of the right cardiophrenic angle at time *k C*_*1,k,*_ and that at time *k ± n C*_*1,k*±*n*_ is calculated. Third, the coordinate in the *y* direction $y_{C_{2,k}\_correct}$ of the corrected left cardiophrenic angle *C*_*2,k_correct*_ is determined to compensate for the relative vertical motion displacement $\vec{d_{k}}$ of the $y_{C_{2,k}\_correct}$ of the corrected left cardiophrenic angle *C*_*2,k_correct*_. Subsequently, the coordinate in the *x* direction $x_{C_{2,k}\_correct}$ of the corrected left cardiophrenic angle *C*_*2,k_correct*_ is determined by calculating the intersection point of the line parallel to the x-axis $y=y_{C_{2,k}\_correct}$ and the left lung field mask edge ${Ι}_{mask\_left\_edge,k}$ . Last, the left hemi-diaphragm *A*_*2,k*_ *C*_*2,k_correct*_ is optimized by a line segment from the corrected left cardiophrenic angle at time *k C*_*2,k_correct*_ to the left costophrenic angle *A*_*2,k*_ along the left lung field mask edge ${Ι}_{mask\_left\_edge,k}$ .

The above specific implementation details are represented by mathematical expressions Equations 9–12:

$$C_{2,k}\left(x,y\right)=C_{2,k\pm n}\left(x,y\right),$$ (9)

$$\vec{d_{k}}=y_{C_{2,k}}-y_{C_{2,k\pm n}},$$ (10)

$$y_{C_{2,k}\_correct}=y_{C_{2,k}}+\vec{d},$$ (11)

$$\left\{\begin{array}{l} y=y_{C_{2,k}\_correct}\\ {Ι}_{mask\_left\_edge,k}=0 \end{array}\right.\to x_{C_{2,k}\_correct}$$ (12)

Where $C_{2,k}\left(x,y\right)$ and $C_{2,k\pm n}\left(x,y\right)$ separately denote the left cardiophrenic angles at time *k* and *k ± n*. Besides, $\vec{d_{k}}$ denotes the relative vertical motion displacement. Furthermore, $y_{C_{2,k}}$ and $y_{C_{2,k\pm n}}$ separately denote the left cardiophrenic angles at time *k* and *k ± n* in the *y* direction. $x_{C_{2,k}\_correct}$ and $y_{C_{2,k}\_correct}$ separately denote the horizontal and vertical coordinates of the corrected left cardiophrenic angle *C*_*2,k_correct*_.

#### 2.2.3 Evaluation metrics

To assess the effectiveness of the proposed method, the standard evaluation metrics in this study include the Euclidean distance error and the length error. The specific evaluation metrics are represented by mathematical expressions Equations 13–18:

$$d_{B_{1,k}\_error}=d\left(B_{1,k},B_{1,k\_GB}\right)=\sqrt{{\left(x_{B_{1,k}}-x_{B_{1,k\_GB}}\right)}^{2}+{\left(y_{B_{1,k}}-y_{B_{1,k\_GB}}\right)}^{2}},$$ (13)

$$d_{C_{1,k}\_error}=d\left(C_{1,k},C_{1,k\_GB}\right)=\sqrt{{\left(x_{C_{1,k}}-x_{C_{1,k\_GB}}\right)}^{2}+{\left(y_{C_{1,k}}-y_{C_{1,k\_GB}}\right)}^{2}},$$ (14)

$$d_{B_{2,k}\_error}=d\left(B_{2,k},B_{2,k\_GB}\right)=\sqrt{{\left(x_{B_{2,k}}-x_{B_{2k\_GB}}\right)}^{2}+{\left(y_{B_{2,k}}-y_{B_{2,k\_GB}}\right)}^{2}},$$ (15)

$$d_{C_{2,k}\_error}=d\left(C_{2,k},C_{2,k\_GB}\right)=\sqrt{{\left(x_{C_{2,k}}-x_{C_{2,k\_GB}}\right)}^{2}+{\left(y_{C_{2,k}}-y_{C_{2,k\_GB}}\right)}^{2}},$$ (16)

$$B_{1,k}C_{1,k}\_error=l_{B_{1,k}C_{1,k}}-l_{B_{1,k}C_{1,k\_GB}},$$ (17)

$$B_{2,k}C_{2,k}\_error=l_{B_{2,k}C_{2,k}}-l_{B_{2,k}C_{2,k\_GB}},$$ (18)

Where $d_{B_{1,k}\_error}$ , $d_{C_{1,k}\_error}$ , $d_{B_{2,k}\_error}$ and $d_{C_{2,k}\_error}$ separately denote the Euclidean distance error of the detected or corrected right and left costophrenic and cardiophrenic angles at time k $B_{1,k}$ , $B_{2,k}$ , $C_{1,k}$ and $C_{2,k}$ . Besides, $B_{1,k\_GB}$ , $B_{2,k\_GB}$ , $C_{1,k\_GB}$ and $C_{2,k\_GB}$ separately denote the ground truth of right and left costophrenic and cardiophrenic angles at time k. $B_{1,k}C_{1,k}\_error$ and $B_{2,k}C_{2,k}\_error$ separately denote the length error of the right and left hemi-diaphragm. In addition, $l_{B_{1,k}C_{1,k}}$ and $l_{B_{2,k}C_{2,k}}$ separately denote the detected or corrected length of the right and left hemi-diaphragm at time k. In addition, $l_{B_{1,k}C_{1,k}\_GB}$ and $l_{B_{2,k}C_{2,k\_GB}}$ separately denote the ground truth of the right and left hemi-diaphragm at time k.

To ensure the consistency and reliability of the ground truths, three radiologists participated in the manual annotation of these ground truths in this study. Specifically, two primary radiologists independently annotate the ground truths on each dynamic CXR image using the software Labelme (v5.1.0). Then, the third experienced radiologist arbitrates or makes final modifications to the disputed annotation results of these ground truths.

#### 2.2.4 Implementation details

The lung field segmentation model is trained on PyCharm 2017.3.3 (Community Edition) in Windows 10 Pro 64-bit, utilizing an NVIDIA GeForce GTX 1080 Ti GPU and 16 GB RAM. Then, the pth format of the lung field segmentation model is converted to the pt format based on PyCharm 2017.3.3. Lastly, the lung field segmentation model with the pt format is called by C++ code based on Visual Studio 2017 for lung field segmentation of the dynamic CXR images of cases 1–5. Similarly, the proposed optimization method is automatically performed for hemi-diaphragm measurement in Visual Studio 2017.

## 3 Results

This section presents the comprehensive results of hemi-diaphragm measurement based on both previous and our proposed optimization methods.

### 3.1 Hemi-diaphragm measurement based on the previous method

**FIGURE 3:**
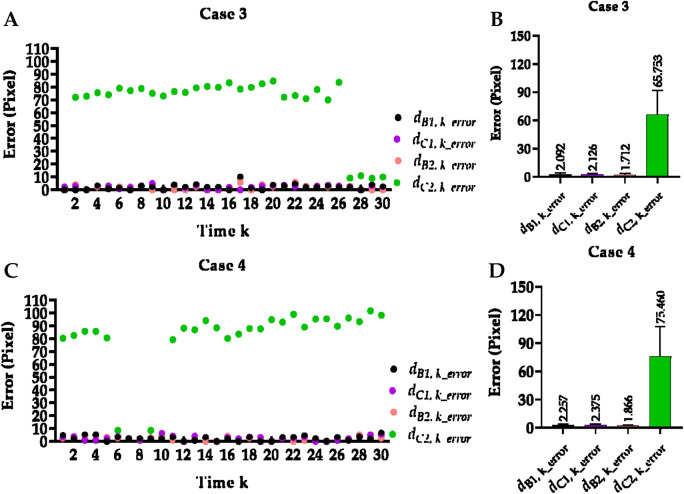
Visual and statistical Euclidean distance error of abnormal left cardiophrenic angles measured by the previous method. **(A)** Visual Euclidean distance error of case 3. **(B)** Statistical Euclidean distance error of case 3. **(C)** Visual Euclidean distance error of case 4. **(D)** Statistical Euclidean distance error of case 4.

**FIGURE 4:**
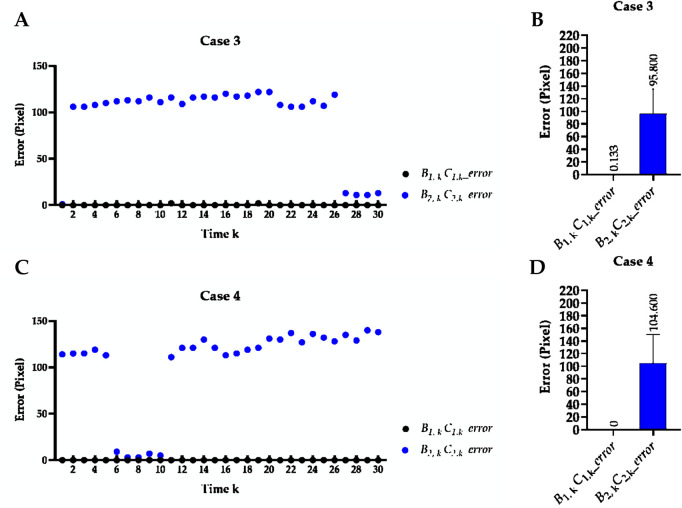
Visual and statistical length error of abnormal left hemi-diaphragms measured by the previous method. **(A)** Visual length error of case 3. **(B)** Statistical length error of case 3. **(C)** Visual length error of case 4. **(D)** Statistical length error of case 4.

Table 2 reports the mean Euclidean distance error of the right and left costophrenic and cardiophrenic angles measured by the previous method (Yang et al., 2024b). Additionally, Figure 3 visually and statistically illustrates these Euclidean distance errors associated with abnormal left cardiophrenic angles. Meanwhile, Table 3 reports the mean length error of the right and left hemi-diaphragms measured by the previous method (Yang et al., 2024b)]. In addition, Figure 4 visually and statistically displays these length errors of abnormal left hemi-diaphragms.

**TABLE 2:** Mean Euclidean distance error of the right and left costophrenic and cardiophrenic angles measured by the previous method.

Case	Mean $d_{B_{1,k}\_error}$	Mean $d_{C_{1,k}\_error}$	Mean $d_{B_{2,k}\_error}$	Mean $d_{C_{2,k}\_error}$	Mean error
1 (30 images)	1.071 ± 1.748 (0.000–5.099)	5.171 ± 2.644 (0.000–9.220)	3.634 ± 1.937 (1.000–8.246)	3.338 ± 2.362 (0.000–10.000)	3.304
2 (30 images)	3.335 ± 1.417 (1.000–6.000)	4.004 ± 2.464 (1.414–11.660)	2.867 ± 2.270 (0.000–8.000)	5.074 ± 2.631 (1.414–14.560)	3.820
3 (30 images)	2.092 ± 2.031 (0.000–10.000)	2.126 ± 1.332 (0.000–5.000)	1.712 ± 1.801 (0.000–6.000)	**65.753 ± 26.420 (2.236–84.853)** ^a^	**17.921** ^a^
4 (30 images)	2.257 ± 1.723 (0.000–6.403)	2.375 ± 1.627 (0.000–6.083)	1.866 ± 1.253 (0.000–5.000)	**75.460 ± 32.631 (1.000–101.820)** ^a^	**20.490** ^a^
5 (30 images)	2.560 ± 1.951 (0.000–8.544)	4.913 ± 2.280 (1.000–8.602)	3.011 ± 1.683 (0.000–7.616)	3.308 ± 3.205 (0.000–17.090)	3.448

^a^ The bold number indicates the outliers of the mean Euclidean distance error.

**TABLE 3:** Length error of the right and left hemi-diaphragms measured by the previous method.

Case	Mean $B_{1,k}C_{1,k}\_error$	Mean $B_{2,k}C_{2,k}\_error$ (pixel)	Mean error
1 (30 images)	1.367 ± 0.964 (0.000–4.000)	1.067 ± 0.9072 (0.000–3.000)	1.217
2 (30 images)	0.933 ± 0.944 (0.000–4.000)	0.900 ± 0.885 (0.000–4.000)	0.917
3 (30 images)	0.833 ± 0.699 (0.000–3.000)	**95.800 ± 39.449 (1.000–122.000)** ^a^	**48.3165** ^a^
4 (30 images)	0.700 ± 0.702 (0.000–2.000)	**104.600 ± 45.874 (3.000–140.000)** ^a^	**52.650** ^a^
5 (30 images)	1.400 ± 0.855 (0.000–3.000)	1.200 ± 0.664 (0.000–3.000)	1.300

^a^ The bold number indicates the outliers of the mean length error.

Specifically, the mean Euclidean distance error of the right and left costophrenic and cardiophrenic angles ( $d_{B_{1,k}\_error}$ , $d_{C_{1,k}\_error}$ , $d_{B_{2,k}\_error}$ , and $d_{C_{2,k}\_error}$ ) of these five cases measured by the previous method is 1.071/3.335/2.092/2.257/2.560, 5.171/4.004/5.759/2.375/4.913, 3.634/2.867/1.712/1.866/3.011, and 3.338/5.074/65.753/75.460/3.308 pixels, respectively. Besides, the mean length error of the right and left hemi-diaphragms ( $B_{1,k}C_{1,k}\_error$ and $B_{2,k}C_{2,k}\_error$ ) of these five cases measured by the previous method is 1.367/1.067, 0.933/0.900, 0.833/95.800, 0.700/104.600, and 1.400/1.200, respectively. Compared with other Euclidean distance errors in Table 3, larger numerical values of Euclidean distance error of the left cardiophrenic angles $d_{C_{2,k}\_error}$ in Figure 3A,C result in the outliers (65.753 ± 26.420 and 75.460 ± 32.63 pixels) of cases 3 and 4. These outliers further contributed to the larger numerical values of the length error of the left hemi-diaphragms $B_{2,k}C_{2,k}\_error$ in Figures 4A,C.

Meanwhile, the times of abnormal and normal left hemi-diaphragms in the 30 dynamic CXR images for each case are also determined, as reflected in Figures 3, 4A,C. For example, except for times 1 and 27–30 of case 3, measurement abnormalities are present in the left hemi-diaphragms at other times. Besides, except for times 6–10 of case 4, there are measurement abnormalities in the left hemi-diaphragms at other times.

### 3.2 hemi-diaphragm measurement optimization

**FIGURE 5:**
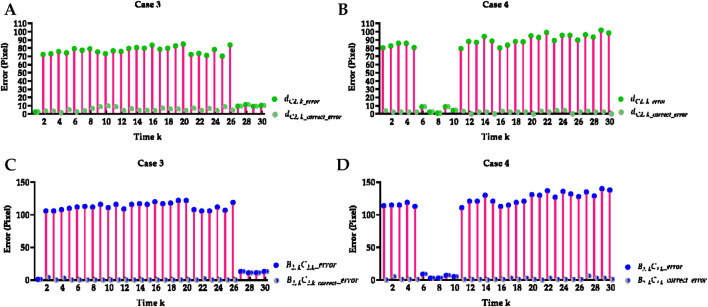
Visual comparison of cases 3 and 4’s Euclidean distance error of left cardiophrenic angles and length error of left hemi-diaphragms measured by the previous and proposed method. **(A)** Visual comparison of case 3’s Euclidean distance error of left cardiophrenic angles measured by the previous and proposed methods. **(B)** Visual comparison of case 4’s Euclidean distance error of left cardiophrenic angles measured by the previous and proposed methods. **(C)** Visual comparison of case 3’s length error of left hemi-diaphragms measured by the previous and proposed methods. **(D)** Visual comparison of case 4’s length error of left hemi-diaphragms measured by the previous and proposed methods.

**FIGURE 6:**
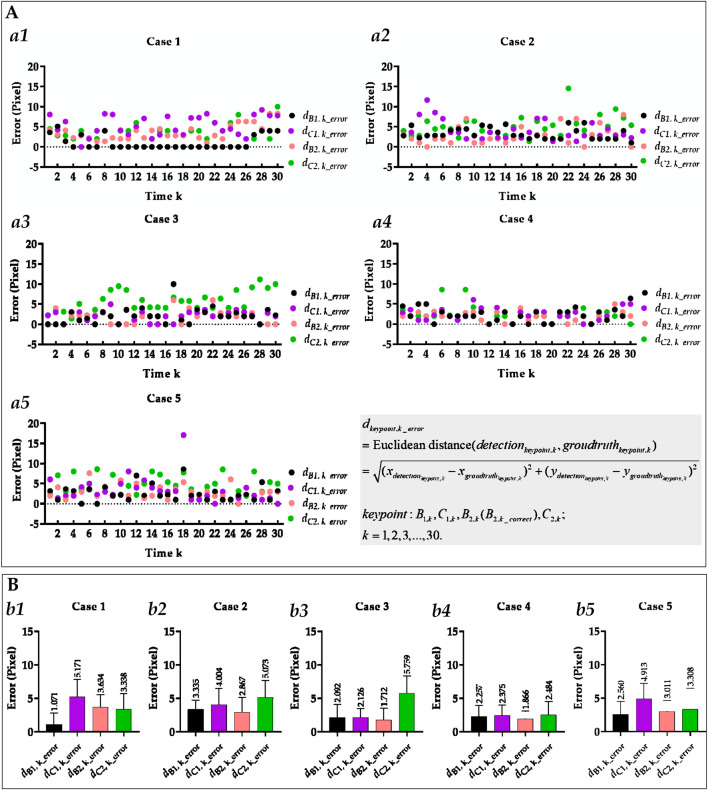
Visual and statistical Euclidean distance error of all costophrenic and cardiophrenic angles measured by the proposed method. **(A)** Visual Euclidean distance error of all costophrenic and cardiophrenic angles of all cases. (a1) Visual Euclidean distance error of all costophrenic and cardiophrenic angles of case 1. (a2) Visual Euclidean distance error of all costophrenic and cardiophrenic angles of case 2. (a3) Visual Euclidean distance error of all costophrenic and cardiophrenic angles of case 3. (a4) Visual Euclidean distance error of all costophrenic and cardiophrenic angles of case 5. (a5) Visual Euclidean distance error of all costophrenic and cardiophrenic angles of case 5. **(B)** Statistical Euclidean distance error of all costophrenic and cardiophrenic angles of all cases. (b1) Statistical Euclidean distance error of all costophrenic and cardiophrenic angles of case 1. (b2) Statistical Euclidean distance error of all costophrenic and cardiophrenic angles of case 2. (b3) Statistical Euclidean distance error of all costophrenic and cardiophrenic angles of case 3. (b4) Statistical Euclidean distance error of all costophrenic and cardiophrenic angles of case 4. (b5) Statistical Euclidean distance error of all costophrenic and cardiophrenic angles of case 5.

**FIGURE 7:**
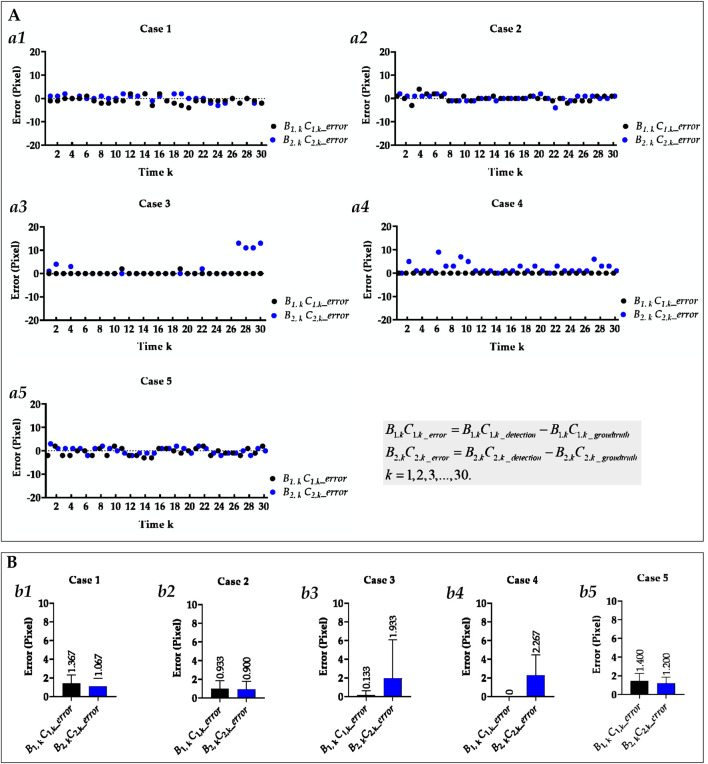
Visual and statistical length error of right and left hemi-diaphragms measured by the proposed method. **(A)** Visual length error of the right and left hemi-diaphragms of all cases. (a1) Visual length error of right and left hemi-diaphragms of case 1. (a2) Visual length error of right and left hemi-diaphragms of case 2. (a3) Visual length error of right and left hemi-diaphragms of case 3. (a4) Visual length error of right and left hemi-diaphragms of case 4. (a5) Visual length error of right and left hemi-diaphragms of case 5. **(B)** Statistical length error of right and left hemi-diaphragms of all cases. (b1) Statistical length error of right and left hemi-diaphragms of case 2. (b2) Statistical length error of right and left hemi-diaphragms of case 2. (b3) Statistical length error of right and left hemi-diaphragms of case 3. (b4) Statistical length error of right and left hemi-diaphragms of case 4. (b5) Statistical length error of right and left hemi-diaphragms of case 5. When statistically displaying the length error of right and left hemi-diaphragms, absolute value calculations were performed on these 30 pairs of length errors to avoid canceling positive and negative errors.

Figure 5 visually displays the comparison of cases 3 and 4’s Euclidean distance error of left cardiophrenic angles and length error of left hemi-diaphragms measured by the previous and proposed method. Table 4 compares the mean Euclidean distance error of the costophrenic and cardiophrenic angles in cases 3 and 4, as measured by both previous and proposed optimization methods. Additionally, Figure 6 visually and statistically illustrates these Euclidean distance errors using our proposed optimization method. Meanwhile, Table 5 compares the mean length error of the right and left hemi-diaphragms in cases 3 and 4, as measured by both previous and proposed optimization methods. Additionally, Figure 7 visually and statistically displays the length errors of the left hemi-diaphragms.

**TABLE 4:** The method comparison of the mean Euclidean distance error of the right and left costophrenic and cardiophrenic angles.

Method	Case	Mean $d_{B_{1,k}\_error}$	Mean $d_{C_{1,k}\_error}$	Mean $d_{B_{2,k}\_error}$	Mean $d_{C_{2,k}\_error}$	Mean error
Yang, et al. [3]	3 (30 images)	2.092 ± 2.031 (0.000–10.000)	2.126 ± 1.332 (0.000–5.000)	1.712 ± 1.801 (0.000–6.000)	65.753 ± 26.420 (2.236–84.853)	17.921
Ours	3 (30 images)	2.092 ± 2.031 (0.000–10.000)	3.472 ± 5.351 (0.000–22.000)	1.712 ± 1.801 (0.000–6.000)	**5.759 ± 2.579 (1.414–11.180)** ^a^↓	**2.922** ^a^↓
Yang, et al. [3]	4 (30 images)	2.257 ± 1.723 (0.000–6.403)	2.375 ± 1.627 (0.000–6.083)	1.866 ± 1.253 (0.000–5.000)	75.460 ± 32.631 (1.000–101.820)	20.490
Ours	4 (30 images)	2.257 ± 1.723 (0.000–6.403)	2.375 ± 1.627 (0.000–6.083)	1.866 ± 1.253 (0.000–5.000)	**2.484 ± 2.041 (0.000–8.602)** ^a^↓	**2.246** ^a^↓

^a^ The bold number indicates better performance compared to the previous method.

**TABLE 5:** The method comparison of the mean length error of the right and left hemi-diaphragms.

Method	Case	Mean $B_{1,k}C_{1,k}\_error$	Mean $B_{2,k}C_{2,k}\_error$	Mean error
Yang, et al. [3]	3 (30 images)	0.833 ± 0.699 (0.000–3.000)	95.800 ± 39.449 (1.000–122.000)	48.317
Ours	3 (30 images)	0.833 ± 0.699 (0.000–3.000)	**1.933 ± 4.143 (0.000–13.000)** ^a^↓	**1.383** ^a^↓
Yang, et al. [3]	4 (30 images)	0.700 ± 0.702 (0.000–2.000)	104.600 ± 45.874 (3.000–140.000)	52.650
Ours	4 (30 images)	0.700 ± 0.702 (0.000–2.000)	**2.267 ± 2.196 (0.000–9.000)** ^a^↓	**1.484** ^a^↓

^a^ The bold number indicates better performanc compared to the previous method.

Specifically, the mean Euclidean distance error of the left cardiophrenic angle measured by the proposed optimization method of case 3 has significantly decreased from 65.753 ± 26.420 (2.236–84.853) to 5.759 ± 2.579 (1.414–11.180) pixels (p-value <0.001), thereby the mean error of all right and left costophrenic and cardiophrenic angles has also significantly decreased from 17.921 to 2.922 pixels (p-value <0.001). Besides, the mean Euclidean distance error of the left cardiophrenic angle measured by the proposed optimization method of case 4 has significantly decreased from 75.460 ± 32.631 (1.000–101.820) to 2.484 ± 2.041 (0.000–8.602) pixels (p-value <0.001), thereby the mean error of all right and left costophrenic and cardiophrenic angles has also significantly decreased from 20.490 to 2.246 pixels (p-value <0.001).

Meanwhile, the mean length error of the right and left hemi-diaphragms measured by the proposed optimization method of case 3 has significantly decreased from 95.800 ± 39.449 (1.000–122.000) to 1.933 ± 4.143 (0.000–13.000) pixels (p-value <0.001), thereby the mean error of the right and left hemi-diaphragms has also significantly decreased from 48.3165 to 1.383 pixels (p-value <0.001). Besides, the mean length error of the right and left hemi-diaphragms measured by the proposed optimization method of case 4 has significantly decreased from 104.600 ± 45.874 (3.000–140.000) to 2.267 ± 2.196 (0.000–9.000) pixels (p-value <0.001), thereby the mean error of the right and left hemi-diaphragms has also significantly decreased from 52.650 to 1.484 pixels (p-value <0.001).

In summary, the mean Euclidean distance error of the left cardiophrenic angle measured by the proposed optimization method of cases 3 and 4 has significantly decreased from 70.606 (≈(65.753 + 75.460)/2) to 4.122 (≈(5.759 + 2.484)/2) pixels (p-value <0.001), thereby the mean error of these right and left costophrenic and cardiophrenic angles has also significantly decreased from 19.206 (≈(17.921 + 20.490)/2) to 2.584 (=(2.922 + 2.246)/2) pixels (p-value <0.001). Besides, the mean length error of the right and left hemi-diaphragms measured by the proposed optimization method of cases 3 and 4 has significantly decreased from 100.200 (=(95.800 + 104.600)/2) to 2.100 (=(1.933 + 2.267)/2) pixels (p-value <0.001), thereby the mean error of these right and left hemi-diaphragms has also significantly decreased from 50.484 (≈(48.317 + 52.650)/2) to 1.434 (≈(1.383 + 1.484)/2) pixels (p-value <0.001). Based on the above, the proposed optimization method can effectively measure the hemi-diaphragm, even in the presence of the inapparent cardiophrenic angle caused by abnormal deformations of the lung field morphology during respiration, reducing the mean error by 49.050 pixels (50.484–1.434).

**FIGURE 8:**
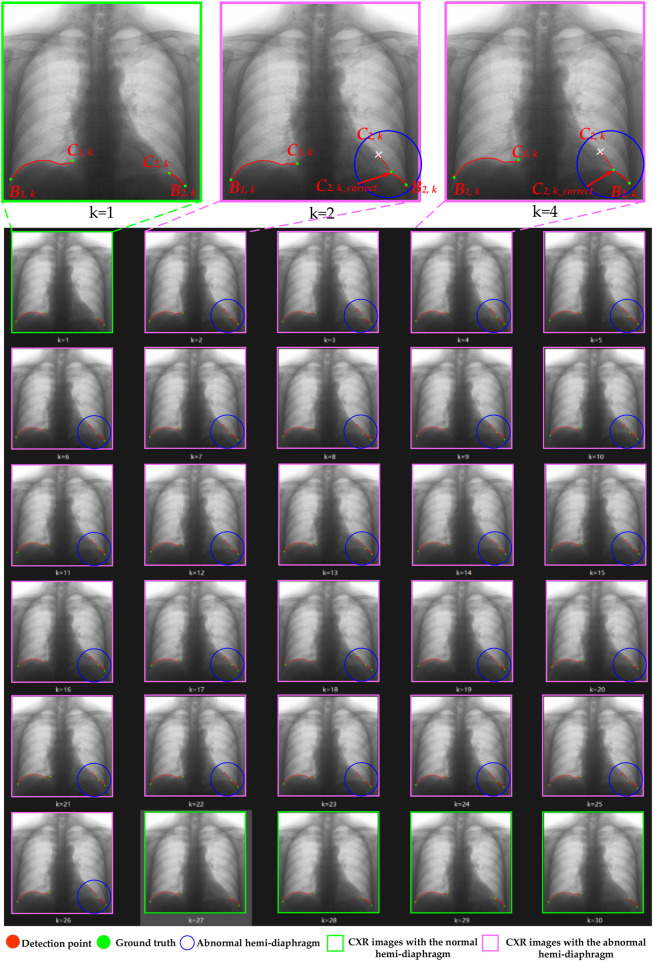
The optimization process of case 3’s left hemi-diaphragm visualizations. The pink box: CXR images with an abnormal hemi-diaphragm that requires correction. The green box: CXR images with the normal hemi-diaphragm.

**FIGURE 9:**
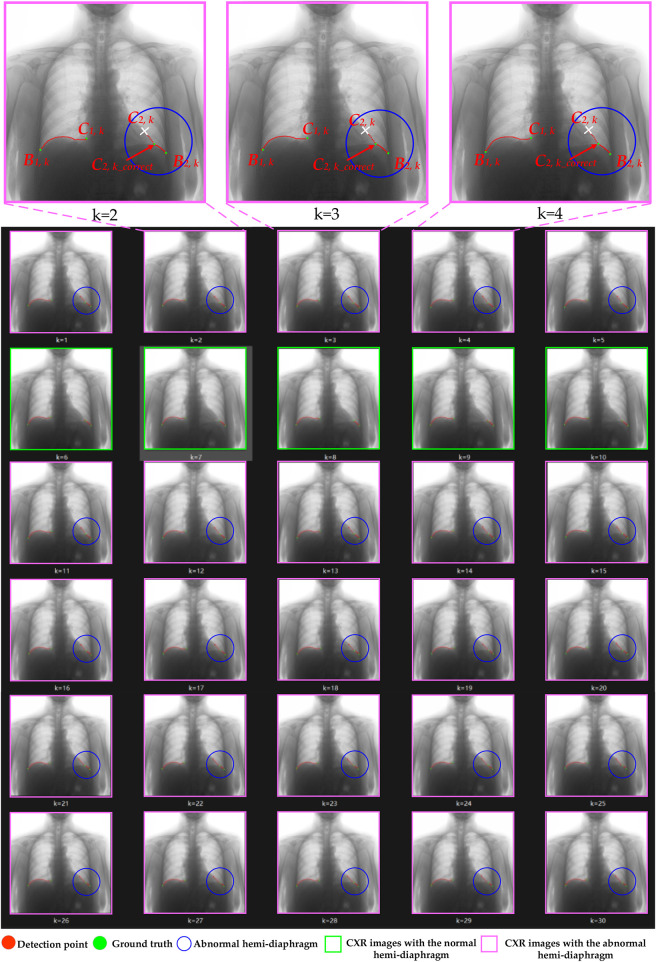
The optimization process of case 4’s left hemi-diaphragm visualizations. The pink box: CXR images with an abnormal hemi-diaphragm that requires correction. The green box: CXR images with the normal hemi-diaphragm.

Figures 8, 9 illustrate the optimization process for the left hemi-diaphragm visualizations of cases 3 and 4. Specifically, Figures 8, 9 display the costophrenic and cardiophrenic angles measured using both the previous and proposed methods on the dynamic CXR images. The dynamic CXR images with abnormal left cardiophrenic angles, as shown in Figures 3, 4, are labeled with pink boxes. Meanwhile, the dynamic CXR images with abnormal left cardiophrenic angles, as shown in Figures 3, 4, are labeled with green boxes.

More specifically, abnormal left cardiophrenic angles *C*_*2,k*_ in the dynamic CXR images of case 3 at the time k = 2–26 are presented with blue circles. Similarly, the abnormal left cardiophrenic angles C2,k in the dynamic CXR images of case 4, at times k = 1–5 and 11–30, are presented with blue circles. Based on our proposed method, these abnormal left cardiophrenic angles, *C*_*2,k*_, are optimized in an orderly manner to the corrected left cardiophrenic angle at time k, *C*_*2,k_correct*_. Each high-resolution image in Figures 8, 9 is provided by the Supplementary Materials (S1_case3 and S1_case4), respectively.

## 4 Discussion

This section discusses the experimental results, highlights the study’s limitations, and suggests future directions.

### 4.1 Morphological deformation of the lung field motion during respiratory interventions

Morphological deformation of the lung field motion during quiet breathing (free respiration or respiratory) interventions is the fundamental reason for abnormal hemi-diaphragm measurement based on the morphology of the lung fields. Lung field segmentation models of dynamic CXR images, abstracted from DCR based on the CNN, can utilize multi-center pathological lung images and data augmentation technology to adapt to morphological deformation changes and achieve satisfactory performance (Yang et al., 2024a; Yang et al., 2024b; Yang et al., 2025). However, these significant deformation changes will increase the risk of abnormal hemi-diaphragm measurement based on the existing method (Yang et al., 2024b).

Specifically, the primary function of the lungs is to facilitate gas exchange between the inspired air and the circulatory system, thereby helping to bring oxygen to the blood and remove carbon dioxide from the body (Jeong et al., 2024). During this respiration process, the lung field motion will undergo morphological deformation. Compared to other static organs or tissues, such as the brain and bones, these morphological deformations of the lung field present a non-rigid and complex state. This non-rigid and complex state is particularly evident in the dynamic CXR images collected during breathing. Additionally, for the dynamic CXR images collected from normal cases during the breathing process, the morphology of the lung field generally does not undergo significant deformation. However, the lungs may suffer from some diseases that can lead to significant deformation changes in lung morphology. For example, end-stage (stage Ⅲ or Ⅳ) chronic obstructive pulmonary disease (COPD) generally causes dyspnea and/or cor pulmonale (Yang et al., 2022a; Yang et al., 2022b). Both dyspnea and/or cor pulmonale caused by COPD often accompany significant deformation changes in the lung field morphology.

### 4.2 Normal and abnormal hemi-diaphragm identification

Identifying normal and abnormal hemi-diaphragms is crucial for optimizing the existing method. Specifically, the abnormal hemi-diaphragm identification aims to determine the hemi-diaphragms that require optimization. In contrast, normal hemi-diaphragm identification assists in optimizing the abnormal hemi-diaphragms, as discussed in the upcoming section on the diaphragm motion consistency criterion.

Additionally, the reasons we configured the shortest hemi-diaphragm among all initial hemi-diaphragms as normal left and right hemi-diaphragms are discussed below. Specifically, the dynamic CXR images are all from a single case, indicating that during the breathing process, if there is no significant change in the captured position, the length of the left and right diaphragm does not increase or decrease significantly. Therefore, this corresponds to the anatomical structure of the diaphragm on the dynamic CXR image. Furthermore, the reason for generating the abnormal hemi-diaphragm is also discussed below. Specifically, the significant deformation changes will shift the cardiac angle upwards on its CXR image, increasing the corresponding initial diaphragm length. Then, the initial hemi-diaphragm measurement requires locating the left and right costophrenic and cardiophrenic angles on the edges of the lung field. However, significant deformation changes in the lung field morphology will result in the cardiophrenic angle not meeting the normal morphological characteristics of the lung field edges at certain moments during the breathing process.

### 4.3 Diaphragm motion consistency criterion

The proposed diaphragm motion consistency criterion is discussed below. Specifically, the primary respiratory muscles involved in respiratory action are the diaphragm, intercostal, and abdominal wall muscles (De Troyer and Moxham, 2020). When inhaling calmly, the diaphragm and intercostal muscles contract, causing an increase in the anterior-posterior, left-right, and up-down diameters of the chest cavity. The lung fields expand accordingly, forming an active inhalation movement. However, when exhaling, the above process is just the opposite.

The diaphragm is the primary respiratory muscle, controlled by the will, that assists with inhalation and expiration (Marufah et al., 2022). During inhalation and respiration, the lower edge of the lung field is uniformly in close contact with the diaphragm. Therefore, the diaphragm’s movement can reflect the lungs’ respiratory process. In anatomy, due to the integrated structure and the mechanical movement of the diaphragm muscle pulling of the left and right hemi-diaphragm, the movement of both the left and right hemi-diaphragm will be carried to the hemi-diaphragm connected to it. Based on the above, we assume that the motion displacement of the cardiophrenic angle of the left and right hemi-diaphragm is consistent within the same time interval, and then optimizes the abnormal cardiophrenic angle using the proposed optimization method. Furthermore, the correction of abnormal hemi-diaphragm is ultimately completed based on the optimized cardiorespiratory angle.

### 4.4 Limitations

Although we propose an optimization method for hemi-diaphragm measurement of DCR that can effectively correct the abnormal hemi-diaphragms, our research still has certain limitations. Specifically, the main limitation is that the proposed optimization method relies on the cardiophrenic angle of the normal hemi-diaphragm to correct the cardiophrenic angle of the abnormal hemi-diaphragm. Therefore, at least an initial normal hemi-diaphragm must be accurately measured based on the two-dimensional morphology of the postero-anterior projection of lung field structure in all dynamic CXR images of DCR (Yang et al., 2024b). Based on the above, once all measured hemi-diaphragms are abnormal, the proposed optimization method will be ineffective. Another limitation is the limited number of DCRs. Therefore, we encourage the collection of various types of diseases and multi-center DCRs from different types of chest X-ray devices to further validate the proposed method, improving the practical applicability and robustness claims.

### 4.5 Perspectives and future work

Although we are committed to proposing a new technology for hemi-diaphragm detection from an engineering perspective for clinical use, further research is needed to evaluate the hemi-diaphragm in different lung diseases or at various stages of the same disease, correlation with clinical endpoints such as the pulmonary function test (e.g., FEV_1_, vital capacity), disease progression, or treatment planning, based on this new technology. In addition, a lung field segmentation model that can input CXR images of multiple sizes needs to be developed to eliminate the errors caused by up-sampling and down-sampling mentioned above. Meanwhile, since the lung field edge image is crucial for optimizing abnormal hemi-diaphragm measurements, novel boundary detection based on CNN should be further proposed (Iqbal et al., 2021; Iqbal et al., 2020; Mochurad, 2025). Additionally, the respiratory intervention in our paper focuses solely on controlling the onset of breathing to collect the DCRs. Furthermore, the pattern of diaphragm movement in other respiratory interventions, such as Yogic breathing, residual efforts, intermittent hypoxia, and voluntary hyperpnea, is expected to be further analyzed based on our proposed method.

## 5 Conclusion

This study proposes an optimization method for hemi-diaphragm measurement based on graphics and the consistency criterion of diaphragm motion, aiming to eliminate the risk of abnormal hemi-diaphragm measurements in existing technologies. Results show that the proposed optimization method can effectively measure the hemi-diaphragm, even in the presence of the inapparent cardiophrenic angle caused by abnormal deformations of the lung field morphology during respiration, reducing the mean error by 49.050 pixels (49.050 × 417 μm = 20,453.85 μm). Therefore, the proposed optimization method may become an effective tool for identifying the pattern of diaphragm movement in respiratory interventions, thereby enhancing precision healthcare.

## Data Availability

The raw data supporting the conclusions of this article will be made available by the authors, without undue reservation.
